# Validation of New Gene Variant Classification Methods: a Field-Test in Diagnostic Cardiogenetics

**DOI:** 10.3389/fgene.2022.824510

**Published:** 2022-03-01

**Authors:** Mohamed Z. Alimohamed, Helga Westers, Yvonne J. Vos, K. Joeri Van der Velde, Rolf H. Sijmons, Paul A. Van der Zwaag, Birgit Sikkema-Raddatz, Jan D. H. Jongbloed

**Affiliations:** ^1^ Department of Genetics, University of Groningen, University Medical Center Groningen, Groningen, Netherlands; ^2^ Department of Haematology and Blood Transfusion, Muhimbili University of Health and Allied Sciences, Dar-es-Salaam, Tanzania; ^3^ Department of Research and Training, Shree Hindu Mandal Hospital, Dar-es-salaam, Tanzania; ^4^ Tanzania Human Genetics Organization, Groningen, Netherlands

**Keywords:** cardiomyopathy, NGS gene panel, variant classification, constraint metrics, cardiogenetics

## Abstract

**Background:** In the molecular genetic diagnostics of Mendelian disorders, solutions are needed for the major challenge of dealing with the large number of variants of uncertain significance (VUSs) identified using next-generation sequencing (NGS). Recently, promising approaches using constraint metrics to calculate case excess scores (CE), etiological fractions (EF), and gnomAD-derived constraint scores have been reported that estimate the likelihood of rare variants in specific genes or regions that are pathogenic. Our objective is to study the usability of these constraint data into variant interpretation in a diagnostic setting, using our cardiomyopathy cohort.

**Methods and Results:** Patients (N = 2002) referred for clinical genetic diagnostics underwent NGS testing of 55–61 genes associated with cardiomyopathies. Previously classified likely pathogenic (LP) and pathogenic (P) variants were used to validate the use of data from CE, EF, and gnomAD constraint analyses for (re)classification of associated variant types in specific cardiomyopathy subtype-related genes. The classifications corroborated in 94% (354/378) of cases. Next, we reclassified 23 unique VUSs to LP, increasing the diagnostic yield by 1.2%. In addition, 106 unique VUSs (5.3% of patients) were prioritized for co-segregation or functional analyses.

**Conclusions:** Our analysis confirms that the use of constraint metrics data can improve variant interpretation, and we, therefore, recommend using constraint scores on other cohorts and disorders and its inclusion in variant interpretation protocols.

## Introduction

The use of next-generation sequencing in molecular diagnostics of Mendelian disorders has improved the diagnostic yield significantly. It allows for testing of an increasing number of genes but, unfortunately, also results in the identification of an increasing number of variants of uncertain significance (VUSs). Moreover, genotype–phenotype associations have not been clearly established for all of the genes analyzed in diagnostics, thereby further contributing to unclear clinical interpretation of genetic variants (Norton et al., 2013; Duzkale et al., 2013; Das et al., 2014; MacArthur et al., 2014). For further interpretation of VUSs, systematic approaches through observations of variant prevalence in larger general population and patient cohorts, co-segregation and/or functional analyses, as well as more sophisticated computational predictions of the potential impact of variants using gene-specific methods are needed (Duzkale et al., 2013; Das et al., 2014; Pugh et al., 2014; Eilbeck et al., 2017; Walsh et al., 2017). However, such data are not sufficiently available for all variants. Therefore, other solutions are needed to further reduce the number of VUSs.

**FIGURE 1:**
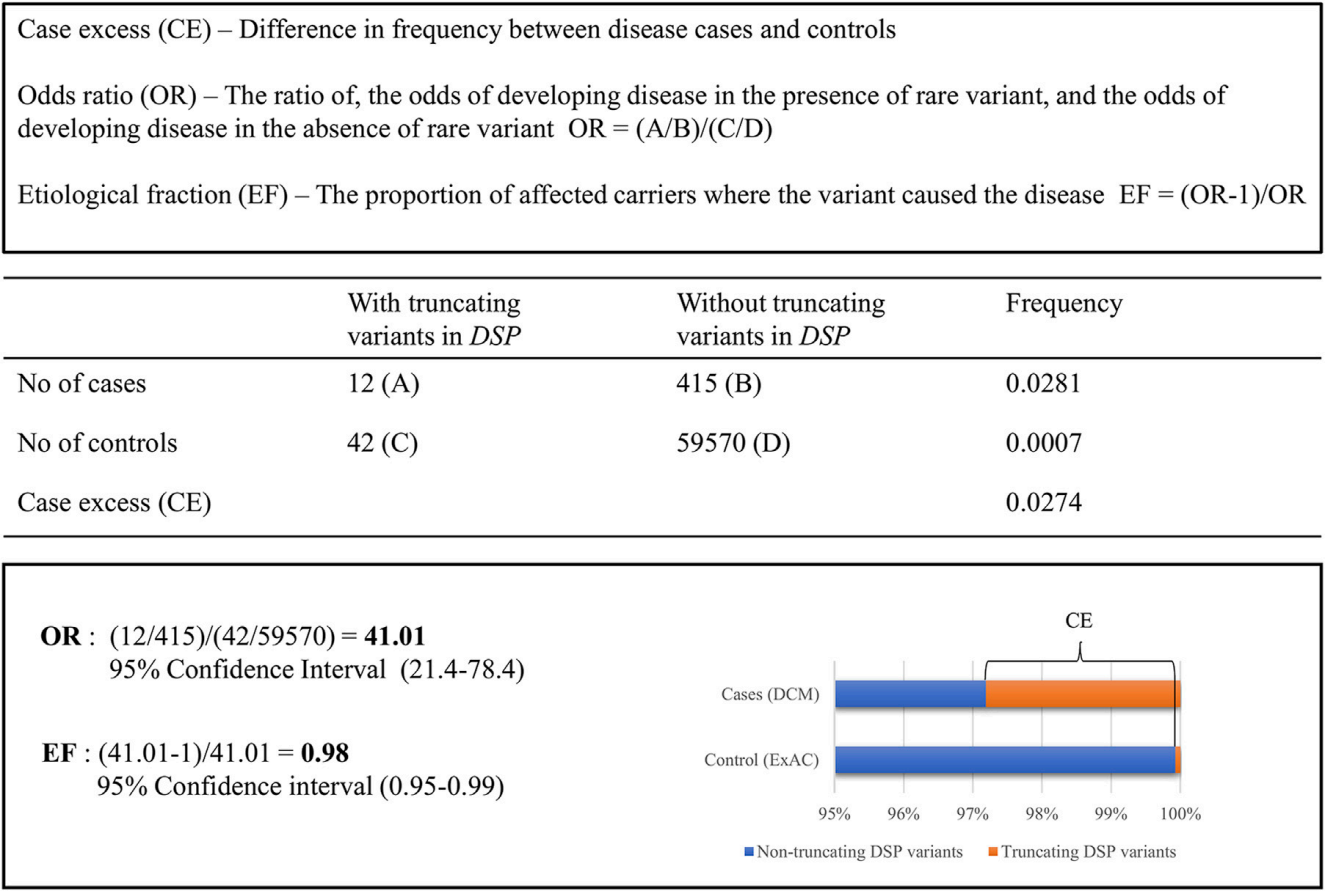
Comparing the frequency of rare truncating variants (ExAC MAF <0.0001) in the *DSP* gene for clinical DCM cases compared to ExAC controls.

Recent studies used constraint metrics to empirically estimate the likelihood that rare variants are pathogenic. The term constraint metrics refers to measures of quantitative assessments leveraging large population genetics data to, for example, compare expected with observed variant frequencies in population cohorts (Lek et al., 2016; Karczewski et al., 2020) or to compare frequencies of rare variations in disease cohorts and that of public databases. Within the field of inherited cardiomyopathies, the latter approach was applied to help the classification of variants identified in cardiomyopathy cases, using allele frequencies published in the exome aggregation consortium (ExAC) database (Walsh et al., 2017; Walsh et al., 2019; Mazzarotto et al., 2020). Case excess (CE) scores and etiological fractions (EFs) were defined as the prior probability that rare variants in the tested cardiomyopathy genes were disease-causing in an affected patient. These CE and EF scores are based on pooled frequency data of rare variants, providing the average risk of variants in a gene being causal and indicating genes associated with particular phenotypes (Walsh et al., 2017) as shown in Figure 1. Indeed, specific variant types (truncating, non-truncating, or both) in established cardiomyopathy genes had clear CE and high EF values (Walsh et al., 2017). In subsequent studies, this method was used to further study established cardiomyopathy subtype specific genes within HCM and DCM (Walsh et al., 2019; Mazzarotto et al., 2020). Moreover, this approach even allowed for the identification of specific regions in HCM genes in which causal variants significantly clustered. Those regions have, therefore, a higher chance of carrying additional variants that are disease causing (Walsh et al., 2019).

Although promising, the outcomes of these statistical methods have not been generally applied in routine clinical diagnostics, and therefore its value in reducing the number of VUSs in such a setting has not been evaluated yet. Our objective was to validate the use of constraint metrics data for variant pathogenicity assessment of cardiomyopathy genes in a diagnostic cardiomyopathy cohort (Alimohamed et al., 2021). We first validated the use of these data for variant classification in a Dutch cohort of 2,002 patients by comparing its outcome to that of variants classified as (L)P applying our routine diagnostic criteria (RDC). Then, we applied constraint scores to interpret VUSs identified in our diagnostics of (established) cardiomyopathy genes and determined whether this may lead to reclassification.

## Materials and Methods

### Patients, Genetic Analysis, and Variant Classification Using Routine Diagnostic Criteria

This study was performed in accordance with UMCG and Dutch national ethical and legal guidelines and complies with the regulations stated in the *Declaration of Helsinki.* Informed consent was obtained for all patients referred to our clinical genetics laboratory.

In total, a consecutive series of 2,002 patients of mainly Caucasian ethnicity from 1,967 families (60.4% male and 39.6% female) were included in our study between 2012 and 2017. Genes known to be implicated in cardiomyopathies were selected for analysis. During the patient inclusion period, three versions of the diagnostic panel were used, the second and third being updated versions. Variants were classified either as “benign” (B)—class 1, “likely benign” (LB)–class 2, “variant of uncertain significance” (VUS)—class 3, “likely pathogenic” (LP)—class 4, or “pathogenic” (P)—class 5. Interpretation was largely based on guidelines recommended by the American College of Medical Genetics and Genomics (ACMG) (Richards et al., 2015). Variants detected in our cohort are routinely submitted to ClinVar (https://www.ncbi.nlm.nih.gov/clinvar). A comprehensive description of the cohort, genetic analysis, variant interpretation, and classification protocols are described in our previous study (Alimohamed et al., 2021).

### Applying Results of Constraint Metric Methods for Prioritization and/or (Re)classification

In recent studies, Walsh and colleagues studied potential CE and EFs of variants identified in cardiomyopathy patients by comparing frequency data of rare variants identified in clinical cardiomyopathy cases with the frequency of these variants in 60,706 ExAC reference samples (Walsh et al., 2017; Walsh et al., 2019, Mazzarotto et al., 2020). Initially, CE was determined for truncating (frameshift, nonsense, and RNA consensus splice donor/acceptor) and non-truncating (missense and in frame insertions and deletions) variants separately for established cardiomyopathy genes in ACM (*N* = 8), HCM (*N* = 20), and DCM (*N* = 48) subtypes (Walsh et al., 2017). In addition, the same group reported higher CE and EF scores in additional studies for DCM (Mazzarotto et al., 2020) and HCM (Walsh et al., 2019). Variant types in genes significantly enriched in patients in these studies are summarized in Supplementary Table S1 and were used for variant interpretation for our cardiomyopathy cohort.

Additionally, for genes in our panel to which the data of the CE/EF-based approach could not be applied, as these genes were not included in the respective analyses, we collected data from an alternative, previously reported approach (Lek et al., 2016; Karczewski et al., 2020) also providing constraint scores. These constraint scores are based on the deviation of observed variant counts from variant counts per gene expected by chance. Variants in genes with a loss of function (LoF) intolerant (pLI) score >0.90 (truncating variants) or a missense (mis_z) score >3 (missense variants) were considered putatively detrimental. Using mis_z and/or pLI scores from the gnomAD database, additional variant types in genes that have an increased risk of being pathogenic were selected. Notably, our procedure of selecting VUSs for prioritization and reclassification based on constraint metric data is also visualized in Supplementary Figure S1.

### Validation: (L)Ps

We initially validated the potential of using constraint scores for prioritizing and (re)classifying variants by determining the number of variants from our cohort classified as LP or P using our routine diagnostic criteria (RDC) (Alimohamed et al., 2021) and the number of these variants that would have been predicted to have increased risk of pathogenicity using the recommendation from the scores from the CE/EF based approach and therefore may be classified (L)P. Next, the concordance between these groups was calculated.

### Prioritization and Reclassification: VUSs

VUSs in genes with significant CE for truncating and/or non-truncating variants within the corresponding cardiomyopathy subtype, as established by Walsh et al., 2017; Walsh et al., 2019; and Mazzarotto et al., 2020, were selected for prioritization for co-segregation and/or functional studies and/or reclassification (see Supplementary Tables 2A,B). The same was done for VUSs following the above criterion, but identified in unclassified CM cases. In addition, for genes in our panel to which the results for the CE/EF based approach could not be applied, VUSs in genes with a pLI>0.90 (LoF variants) or a mis_z > 3 (missense/in frame del/dup) were also selected (Supplementary Table S3). Then, depending on the respective EF scores of the corresponding variant types in the thus previously analyzed genes, VUSs were either immediately reclassified to LP (EF ≥ 0.90) or prioritized for further analyses/studies (EF < 0.90) (Lek et al., 2016; Karczewski et al., 2020) such as co-segregation analyses or functional studies, potentially leading to reclassification. In addition, the latter was also applied to VUSs in genes selected on the basis of the gnomAD-based constraint metrics (pLI>0.9 or mis_z > 3.0).

## Results

### Correlation Between Classification *via* Routine Diagnostic Criteria and Classification Using Results of Constraint Metrics-Based Statistical Methods

In our cohort of 2,002 patients, the overall molecular diagnostic yield, defined as patients carrying at least one LP or P variant following RDC in genes with established associations with the patient’s phenotypes, was 21.5% (430/2002). From the 430 patients carrying these (L)P variants, 378 in 369 patients (note that a few patients carry more than one (L)P) were found in genes analyzed within DCM, HCM, or ACM cohorts (see Table 1 for total and subtype-specific numbers). Of these 378 variants, 354 would have been classified as (L)P on the basis of case excess (CE) in patient cohorts, showing a 94% (354/378) concordance between our RDC- and the CE-based classification. Of the remaining 6% of (L)P variants that were not concordant, 4% were Dutch founder mutations (Alimohamed et al., 2021). In addition, when analyzing genes not included in the aforementioned evaluation, 22 patients carrying 22 (L)Ps in these genes were identified. When using the LoF and missense intolerance scores as provided by the gnomAD database (Lek et al., 2016; Karczewski et al., 2020) for classification, 12 of these would have been classified as (L)P showing a concordance of 55% (12/22).

**TABLE 1:** Impact of retrospective reassessment of variant pathogenicity. **(A)** Reclassification/prioritisation of variants according to CE/EF scores. **(B)** Prioritization of variants in genes with mis_z > 3 or pLI >0.9 according to gnomAD.

**(A)** Reclassification/prioritisation of variants according to CE/EF scores.								**(B)** Prioritization of variants in genes with mis_z > 3 or pLI >0.9 according to gnomAD.
CM Subtype	No. of genes with CE	No. of patients with LP/P variants according to RDC (no. of variants)	(i)		(ii) *Analyses of VUS*		No. of patients without genetic diagnosis, but variants (VUS) leading to definitive reclassification to LP	No. of patients
			No of patients with LP/*p* variants according to CE/EF scores (no. of variants)	Correlation of variant classification between RDC and CE/EF scores	No. of patients with prioritised variants because of CE	No. of patients without genetic diagnosis, but variants possibly leading to reclassification		
HCM	14	169 (172)	158 (160)	93%	61	44	19	—
DCM	14	181 (186)	178 (177)	95%	55	46	6	2
ACM	5	19 (20)	17 (17)	85%	1	1	—	—
CM	—	—	—	—	26	22	—	11
LVNC	—	—	—	—	—	—	—	6
TOTAL	33	369 (378)	353 (354)	94%	143	113	25	19
No. of patients with selected variants	—	—	—	—	113 (6%)		25 (1.2%)	19 (1%)

A) 1) Variants classified as (L)P following RDC, CE/EF criteria and their correlations, 2) patients identified, after application of CE/EF scores on VUSs in CM subtype-specific genes, with reclassified variants and/or variants prioritized for future functional and/or co-segregation studies. B) Additional VUSs prioritized of genes not yet addressed within the CE/EF approach and having significant missense Z (mis_z) and/or probability of loss-of-function intolerant (pLI) scores adapted from the genome aggregation (gnomAD) database.

### Selection of VUSs for Prioritization/Reclassification

VUSs were selected based on published constraint metric data and consisted of three sets: 1) those identified in genes showing CE in patients with specific CM subtypes (HCM, DCM, and ACM), 2) those identified in the same genes but found in patients with unclassified CM where such phenotype matching was not possible, and 3) those identified in genes not having established associations with the patient’s subtypes according to the Walsh and Mazzarotto methods [2017; 2019; 2020] (including those in patients with unclassified CM) and having pLI>0.90 or mis_z > 3. In genes with significant CE for non-truncating and/or truncating variants, a total of 92 unique VUSs in 143 patients were identified in DCM (*N* = 55), ACM (*N* = 1), CM (*N* = 26), and HCM (*N* = 61) to qualify for prioritization/reclassification (Supplementary Tables 2A,B). Of these, 113 patients (46 DCM, 44 HCM, 1 ACM, and 22 CM) did not have a genetic diagnosis yet (the other 30 patients did already carry at least one (L)P), accounting for 5.6% of the total cohort (Table 1).

Next, in genes not included in our previous approach, variants in genes intolerant to variation, having a pLI>0.9 or mis_z > 3.0 score, according to gnomAD v2 aggregate data, were selected (Lek et al., 2016; Karczewski et al., 2020). The resulting variants were prioritized only when identified in genes established to be associated with the patient’s cardiomyopathy subtype (37 different VUSs in 42 patients; Supplementary Table S3). This resulted in the identification of additional 19 patients (2 DCM, 6 LVNC, and 11 CM) that did not have a genetic diagnosis yet, accounting for 1% of the total cohort (Table 1).

### Reclassification and Prioritization of VUSs

As described above, 129 unique variants previously classified as VUS were selected for reclassification and/or prioritization for further analyses. The potential to use CE/EF scores for prioritization/reclassification was underscored by the fact that high scores of pathogenicity prediction programs, often in combination with (very) low population frequencies of these variants, were found for these variants (Supplementary Table S2A). Based on their analyses of CE and EF of HCM-related genes, Walsh et al. [2019] suggested including EF scores in the generally used ACMG guidelines for variant classification. This would result in the adaptation of criterion PM1 (a mutational hot spot or well-defined functional domain without a benign variation), into PM1_supporting (a non-truncating variant in a gene or protein with 0.8 ≤ EF < 0.9), PM1_moderate (a non-truncating variant in a gene or protein with 0.9 ≤ EF < 0.95), and PM1_strong (non-truncating variants a in gene or protein region with EF ≥ 0.95). We decided to implement these adaptations to our classification criteria and expanding this also to truncating variants, within the respective subtype for which the EF was determined. Also, in addition, these genes/variants were identified in unspecified CM cases. Most of the selected VUSs do also fulfill the (*MYH7-*adpated—in our opinion these criteria can be generally applied for every established, autosomal dominant-inherited cardiomyopathy gene) ACMG criterion PM2 (absent/extremely rare (<0.004%) from large population studies) (note that those with population frequencies >0.004% were not considered for reclassification, such as *MYH7* c.2890G > C, p.Val864Leu), and all fulfilled PP3 (multiple lines of computational evidence support a deleterious effect on the gene or gene product) and PP4 (patient’s phenotype or family history is highly specific for a disease with a single genetic etiology) (Richards et al., 2015; Kelly et al., 2018). Following the rules for combining criteria to classify sequence variants as proposed by the ACMG (Richards et al., 2015), this would mean that combining these with the adapted criteria PM1_strong or PM1_moderate would result in 23 unique VUSs in 28 patients being immediately reclassified to LP as these fulfill rule 2) 1 strong and 1–2 moderate, rule 3) 1 strong and ≥2 supporting, and/or rule 5) 2 moderate and ≥2 supporting. Notably, for some cases this applies to variants in specific clusters only, as determined for sarcomeric HCM genes (Walsh et al., 2019), while for others this would apply to variants throughout the gene. Reclassification of these VUSs to LP is substantiated by further proof for pathogenicity as provided in Supplementary Table S2B, of which most would lead to applying ACMG criteria PS4 or adaptations thereof. Due to this reclassification, 25 of these 28 patients [3 patients already carry another (L)P] now also obtain a genetic diagnosis. This results in an increase in the diagnostic yield by 1.2%. The other selected VUSs with CE but EF < 0.9 and/or VUSs with pLI>0.9 or mis_z > 3.0 were prioritized for further analyses such as co-segregation and haplotype-sharing analyses or functional evaluations.

## Discussion

In this study, we applied data from previously published statistical approaches that identified genes and variant types having a higher chance of being disease associated to reduce the amount of VUSs identified during clinical genetic diagnostics, using the example of inherited cardiomyopathies. As a result, we were able to reclassify gene variants from VUS to LP in 25 patients, applying an EF of ≥0.90 leading to an 1.2% definitive increase in the molecular diagnostic yield. Moreover, this resulted in a further 5.3% potential increase in the yield of VUSs prioritized for additional analyses such as co-segregation and functional studies. Important to note is that our results confirm the relevance of the used outcome of statistical methods for gene variant classification, as 94% of variants previously classified as (L)P would also have been classified as such using the constraint rules. Together, applying these rules in daily practices will lead to more diagnoses, as well as guide further analysis of potentially causal VUSs.

With current population data, applying the CE/EF criteria (Walsh et al., 2019) in our cohort resulted in identifying up to 6.6% of additional patients with a potential LP/P reclassification within different CM subtypes. Our results are comparable to those reported by Walsh et al. (2019), where 4% of actionable variants in HCM cases were upgraded to LP. The more general use of these CE/EF scores is consistent with the use of recently established guidelines for *MYH7*-associated inherited cardiomyopathies (Kelly et al., 2018). First, the use of these scores as one of the criteria for classification is comparable with applying the *MYH7*-adapted rule PS4. The rule is that the prevalence of the variant in affected individuals is significantly increased compared with the prevalence in controls—OR—that the variant is identified in ≥15 probands with consistent phenotypes, the difference being that we do apply the CE/EF scores to all potentially causal VUSs within genes for which these were established, while the PS4 criterion in Kelly et al. is applied at the level of individual variants only. Importantly, we only reclassify variants in genes with EF ≥ 0.9 and criteria PM2 (absent/extremely rare (<0.004%) from large population studies) was met, as was also required for using the *MYH7*-adapted PS4 criteria (Kelly et al., 2018). In addition, the fact that higher EF scores could be applied to variants in specific clusters in sarcomeric genes is consistent with the use of the adapted *MYH7* rule PM1 [hotspot/est. functional domain (amino acids 181–937 without benign variation)], however, now underscored with data from CE analyses for *MYH7* and extended to other (sarcomeric) genes (Kelly et al., 2018). Finally, in a situation where a gene associated with the respective cardiomyopathy subtype is having an EF ≥ 0.9, we thus support the view to circumvent the need to conduct functional studies for these cases as previously described (Roca et al., 2018).

Our reclassifications also resulted in three patients that already had a genetic diagnosis now being carrier of two (L)Ps, instead of only one, related to their phenotype. Moreover, prioritized VUSs were identified in an additional 23 previously “solved” cases, and these patients are potentially carrier of multiple (L)Ps related to their disease. This will have significant effects on the management of the respective patients and their family members. In particular, for family members that were previously shown not to carry the already known (L)P and for that reason were dismissed of regular follow-up.

We decided that variant classes with EF scores of <0.90 should not directly be reclassified to LP in a clinical diagnostic setting. This is consistent with the ACMG open forum consensus (Richards et al., 2015) agreeing with a 0.90 cut off value to recognize a variant as LP, while the International Agency for Research on Cancer (IARC) guidelines embraces 0.95 (Richards et al., 2015). We thus agree with suggestions to use EF ≥ 0.95 as strong evidence to reclassify VUSs to LP and EF ≥ 0.90 < 0.95 as moderate evidence. However, identifying genes with variants with CE, but EF < 0.90, as well as genes with pLI>0.9 or mis_z > 3.0 can help in prioritizing these for follow up like co-segregation or functional analyses, in our case 129 unique VUSs in 132 patients from our cohort (6.6%). Moreover, when *MYH7-*adapted co-segregation-based criteria (PP1_strong; variant segregates with ≥7 meioses, PP1_moderate; variant segregates with ≥5 meioses, or PP1_supportive; variant segregates with ≥3 meioses) is more generally applied to other cardiomyopathy genes, having a variant segregating with disease in only a limited number of family members could already lead to upgrading a variant in a gene with CE but EF < 0.9, or a gene with pLI>0.9 or mis_z > 3.0 to an LP status.

Additional studies need to validate whether the data of population-based statistical methods are sufficient for definitive reclassification of such variants for scores between 0.8 ≤ EF < 0.9. Moreover, to attain EF ≥ 0.90 for all relevant genes, if possible at all, more studies are needed to reach higher EFs or identify specific regions within a gene that carry pathogenic variants using larger cohorts for specific cardiomyopathy subtypes. This underscores the complementarity of EF with machine-learning (ML)-based variant pathogenicity predictors such as CADD, CAPICE, and the like (Rentzsch et al., 2019; Li et al., 2020), which present part of the evidence to classify variants as (L)P in novel regions that lack CE/EF ≥ 0.90, or help reclassify variants in known CE/EF ≥ 0.90 regions, as we have shown 94% of variants classified using RDC are concordant to the CE/EF method. When sufficient ‘critical mass’ of pathogenic classifications and population variants becomes available in a region of interest, a high EF may be established for a particular variant type (i.e., truncating variants). This criterion may then be used as a rapid and straightforward variant classifier for unseen variants, perhaps supported by an ML predictor as second opinion or safety net. When available, we recommend starting the variant classification process for selected genes by first looking at CE/EF values as initial criterion. Moreover, as using these values shows to complement the current variant interpretation framework, we therefore propose that including CE/EF classifiers in a variant interpretation framework would benefit currently used interpretation and classification approaches. When applied to other disease genes implicated in Mendelian diseases, this framework offers the potential to generally increase diagnostic yield in genetic testing.

## Limitations

For genes with no significant CE and only computational proof for disease association, there is currently insufficient evidence from the constraint scores methods that a variant will be disease causing. Extended CE and/or clustering analyses may establish their association with disease enabling the use of such constraint scores for variant classification or actually refute disease association. In this respect, it is important to note that no variant classes in the cardiomyopathy genes presented significant depletion in cardiomyopathy cases compared to the gnomAD aggregate database (Walsh et al., 2017), and these variants were therefore not considered for provisional reclassification to LB. Notably, additional research is needed to further validate whether the use of constraint scores with 0.8 ≤ EF < 0.9 for variant interpretation is sufficient for definitive classification of (L)P variants to P or prioritized VUSs to (L)P. To ultimately classify the latter variants as LP or P, further segregation analysis or functional evidence is needed. Moreover, we have only screened for variants in coding regions and surrounding regions of interest, ±20 bases, in ∼60 selected cardiomyopathy genes. Deep intronic or regulatory (5′and 3′UTR) variants and variants in novel genes with potential functional roles in cardiomyopathies were not included in our analysis. Also, as not all genes in our panels have been analyzed for CE and EF, for those we relied on gnomAD-derived constraint scores for determining their putative causal nature, leading to prioritization for further analyses only. The latter because we felt that the strength of these scores were insufficient to support immediate (L)P classification. This was also underscored by the fact that using these constraint scores only 55% of RDC derived (L)Ps in the respective variant types and genes would have been classified as (L)P using these scores. Finally, patient phenotypic information was obtained from referral forms and not scrutinized according to definitive phenotypic criteria.

## Conclusion

Applying CE scores and EF (i.e., constraint metrics)-based evaluations confirmed 94% of classified (L)P variants compared to RDC in a cohort of patients with cardiomyopathy, underscoring the fact that such scores can be used to complement variant interpretation and classification methods. Most importantly, it led to a 1.2% definitive increase (VUSs reclassified to LP) and 5.3% relative increase (VUSs prioritized) in actionable variants in our cardiomyopathy cohort. In addition, using the constraint metrics helped select 37 unique variants in genes with etiological fractions<0.9, or mis_z > 3 or pLI>0.9 using gnomAD constraint data for future co-segregation studies and functional assays. Our analysis underscores that the use of such constraint metrics scores can improve variant interpretation and we recommend validating this method in other cohorts and disorders and consider its inclusion in variant interpretation protocols and implement this for cardiomyopathies.

## Data Availability

The original contributions presented in the study are included in the article/Supplementary Material, further inquiries can be directed to the corresponding authors.
