# Supplementary material for: Validation of New Gene Variant Classification Methods: a Field-Test in Diagnostic Cardiogenetics
Source: Front Genet. 2022 Mar 1;13:824510. doi: 10.3389/fgene.2022.824510 (PMC8921548; doi:10.3389/fgene.2022.824510)
Supplement: Supplementary file 1 [file DataSheet1.docx]

**Validation of new gene variant classification methods: a field-test in diagnostic cardiogenetics**

Mohamed Z. Alimohamed^1,2,3,4*^, Helga Westers^1^, Yvonne J. Vos^1^, K. Joeri van der Velde^1^, , Rolf H. Sijmons^1^, Paul A. van der Zwaag^1^, Birgit Sikkema-Raddatz^1ǂ^, Jan D. H. Jongbloed^1ǂ*^

^1^University of Groningen, University Medical Center Groningen, Department of Genetics, Groningen, the Netherlands

^2^Department of Haematology and Blood Transfusion, Muhimbili University of Health and Allied Sciences, Dar-es-Salaam, Tanzania

^3^Department of Research and Training, Shree Hindu Mandal Hospital, Dar-es-salaam, Tanzania

^4^Tanzania Human Genetics Organization

^ǂ^ Authors contributed equally to this work

*Corresponding authors: Mohamed Z. Alimohamed and Jan D.H. Jongbloed University of Groningen, Department of Genetics, HPC CB50, University Medical Center Groningen, P.O.Box 30001, 9700 RB, Groningen; phone: +31-503617100, fax: +31-503617231, email: m.z.a.alimohamed@umcg.nl, j.d.h.jongbloed@umcg.nl

**Supplemental material**

**Contents**

1. **Supplemental Tables**

Supplemental Table 1. Genes per cardiomyopathy subtype reported with case excess

Supplemental Table 2a. VUSs in genes/gene regions with CE selected for prioritisation within specific CM subtypes in our patient cohort

Supplemental Table 2b. VUSs in genes/gene regions with EF≥0.9 and reclassified to LP within specific CM subtypes in our patient cohort

Supplemental Table 3. VUSs detected in genes with mis_z>3 and pLI>0.9 according to gnomAD

1. **Supplemental Figure**

Supplemental Figure 1. VUS classification/prioritisation flow chart

**Supplemental Table 1. Genes (per cardiomyopathy subtype) with reported case excess**

| Phenotype | **Gene** | **Non-truncating** | **Truncating** |
| --- | --- | --- | --- |
| **HCM** | *ACTC1* | 2 (0.88*; 0.88^#^) |  |
|  | *CSRP3* | 5 (0.69^#^ 0.88^#2^) | 1 (0.84^#^) |
|  | *FHL1* | 0 (0.86*; 0.89^#^) | 0 (>0.99^#^) |
|  | *GLA* | 2 (0.86*) |  |
|  | *MYBPC3* | 7 (0.82*; 0.88^#^ 0.98^#2^) | 0 (0.99*^,#^) |
|  | *MYH7* | 19 (11,4) (0.92*; 0.93^#^ 0.98^#2^) |  |
|  | *MYL2* | 0 (0.85*; 0.89^#^) |  |
|  | *MYL3* | 2 (1) (0.80*; 0.83^#^ 0.93^#2^) |  |
|  | *PLN* | 0(0.85^#^) | 0 (0.96*^,#^) |
|  | *PRKAG2* | 1 (0.49*) |  |
|  | *TNNC1* | 2 (0.77^#^) |  |
|  | *TNNI3* | 0 (0.91*; 0.93^#^ 0.97^#2^) | 0 (0.88^#^) |
|  | *TNNT2* | 2(1,1) (0.88*; 0.91^#^ 0.96^#2^) | 0 (0.83*; 0.84^#^) |
|  | *TPM1* | 1 (0.94*; 0.95^#^) |  |
|  |  |  |  |
| **DCM** | *ACTC1* | 0 (0.86^) | 0 (>0.86^) |
|  | *BAG3* |  | 0 (>0.99^) |
|  | *DSP* |  | 0 (0.98*; 0.97^) |
|  | *LMNA* | 7 (0.79*; 0.74^) | 0 (0.99*; >0.99^) |
|  | *MYH7* | 19 (0.75*; 0.70^) |  |
|  | *NEXN* | 8 (0.75^) | 4 (0.75^) |
|  | *PLN* | 0 (0.89^) | 0 (0.89^) |
|  | *SCN5A* |  | 0 (0.94*) |
|  | *TCAP* | 0 (0.82*) |  |
|  | *TNNC1* | 2 (0.97*; 0.96^) |  |
|  | *TNNT2* | 2 (0.93*; 0.94^) |  |
|  | *TPM1* | 2 (0.95*; 0.96^) |  |
|  | *TTN* |  | 2 (0.95*; 0.96^) |
|  | *VCL* |  | 0 (0.95*; 0.96^) |
|  |  |  |  |
| **ACM** | *DSC2* |  | 0 (0.95*) |
|  | *DSG2* | 0 (0.65*) | 0 (0.95*) |
|  | *DSP* | 1 (0.52*) | 0 (0.99*) |
|  | *JUP* | 0 (0.87*) |  |
|  | *PKP2* |  | 0 (1.00*) |

Truncating mutations: nonsense, frameshift, RNA consensus splice donor/acceptor; Non truncating mutations: missense and in frame deletions and insertions.

For every gene with case excess the number of VUSs, in patients with the respective phenotype, found in this study is indicated, as well as (in brackets) the respective EF as reported by Walsh et al., 2017*, Walsh et al, 2019^#^, Walsh et al, 2019 HCM cluster^#2^ and/or Mazzarotto et al., 2020^. EF values 0.8≤EF<0.9are represented in green and indicate PM1_supporting pathogenic. EF values 0.9≤EF<0.95are represented in orange and indicate PM1_moderate pathogenic. EF values ≥0.95 are represented in red and indicate PM1_strong pathogenic. VUS representing PM1_moderate and PM1_strong are reclassified to LP. *TTN* variants colored in blue are prioritised but not reclassified to LP as LoF mutations in exon 51 up to 219 of this gene are by definition not considered disease causing.

**Supplemental Table 2a. VUSs in genes/gene regions with CE selected for prioritisation within specific CM subtypes in our patient cohort**

| **Variant** | **Protein change** | **Cases/VUS** | **AC; AF(%) gnomAD** | **Path pred** |
| --- | --- | --- | --- | --- |
| *LMNA* c.71C>T | p.(Thr24Ile) | 1(1) | 0 | Ala:3/4; CADD: 25.1(23.7); Var: LP |
| *LMNA* c.232A>G | p.(Lys78Glu) | 1(1) | 0 | Ala:4/4; CADD: 28.2(23.7); Var: P |
| *LMNA* c.398G>A | p.(Arg133Gln) | 1(1) | 3; 0.0023 | Ala:3/4; CADD: 25.6(23.7); Var: P |
| *LMNA* c.437C>A | p.(Ala146Asp) | 1(1) | 0 | Ala:4/4; CADD: 28.7(23.7); Var: P |
| *LMNA* c.467G>T | p.(Arg156Leu) | 1(0) | 0 | Ala:4/4; CADD: 27.8(23.7); Var: LP |
| *LMNA* c.599T>G | p.(Met200Arg) | 1(1) | 0 | Ala:3/4; CADD: 24.2(23.7); Var: P |
| *LMNA* c.647G>A | p.(Arg216His) | 1(0) | 5; 0.013 (Other) | Ala:2/4; CADD: 28.6(23.7); Var: LP |
| *LMNA* c.1001G>A | p.(Ser334Asn) | 1(1) | 6; 0.0039 | Ala:1/4; CADD: 22.4(23.7); Var: VUS/LP |
| *LMNA* c.1280G>A | p.(Arg427His) | 1(1) | 1; 0.00078 | Ala:0/4; CADD: 23.8(23.7); Var: VUS/LP |
| *MYH7* c.38C>T | p.(Ala13Val) | 1(1) | 0 | Ala:3/4; CADD: 23.9(22.7); Var: VUS/LP |
| *MYH7* c.328G>A | p.(Gly110Ser) | 1(1) | 4; 0.0054 (East Asian) | Ala:0/4; CADD: 14.8(22.7); Var: VUS |
| *MYH7* c.784G>A | p.(Asp262Asn) | 1(1) | 0 | Ala:1/4; CADD: 23.8(22.7); Var: VUS/LP |
| *MYH7* c.1263A>G | p.(Ile421Met) | 1(1) | 0 | Ala:1/4; CADD: 0.9(22.7); Var: VUS |
| *MYH7* c.1640C>G | p.(Thr547Ser) | 1(1) | 0 | Ala:4/4; CADD: 21.8(22.7); Var: VUS |
| *MYH7* c.2890G>C | p.(Val964Leu) | 6(6) | 124; 0.084 | Ala:4/4; CADD: 26.9(22.7); Var: VUS/LP |
| *MYH7* c.3286G>T | p.(Asp1096Tyr) | 1(1) | 40; 0.031 | Ala:3/4; CADD: 32.0(22.7); Var: LB |
| *MYH7* c.4084T>C | p.(Ser1362Pro) | 1(1) | 0 | Ala:3/4; CADD: 26.2(22.7); Var: VUS/LP |
| *MYH7* c.4377G>T | p.(Lys1459Asn) | 2(2) | 83; 0.053 | Ala:3/4; CADD: 26.2(22.7); Var: VUS/LP |
| *MYH7* c.4750G>A | p.(Glu1584Lys) | 1(1) | 0 | Ala:3/4; CADD: 28.4(22.7); Var: VUS |
| *MYH7* c.4996G>A | p.(Asp1666Asn) | 1(1) | 2; 0.0017 | Ala:3/4; CADD: 30.0(22.7); Var: VUS/LP |
| *MYH7* c.5174A>G | p.(Asn1725Ser) | 1(1) | 0 | Ala:3/4; CADD: 24.1(22.7); Var: VUS/LP |
| *MYH7* c.5401G>A | p.(Glu1801Lys) | 1(1) | 0 | Ala:3/4; CADD: 31.0(22.7); Var: P |
| *MYH7* c.5470_5471delinsGG | p.(Asn1824Gly) | 1(0) | 1; 0.00088 | Ala:3/4; CADD: 27.9(22.7); Var: VUS |
| *NEXN* c.157G>A | p.(Glu53Lys) | 2(2) | 11; 0.0082 (African/African American) | Ala:2/4; CADD: 28.7(35.2); Var: B |
| *NEXN* c.380G>A | p.(Arg127His) | 1(0) | 7; 0.027 (East Asian) | Ala:2/4; CADD: 28.9(35.2); Var: B |
| *NEXN* c.1174T>C | p.(Arg392*) | 4(4) | 7; 0.0046 | Ala:n.a.; CADD: 38.0(35.2); Var: B |
| *NEXN* c.1453G>A | p.(Gln485Lys) | 3(3) | 15; 0.014 (Other) | Ala:3/4; CADD: 33.0(35.2); Var: B |
| *NEXN* c.1996A>G | p.(Thr666Ala) | 1(1) | 9; 0.014 (Other) | Ala:4/4; CADD: 22.9(35.2); Var: B |
| *NEXN* c.1350C>T | p.(Ser450Ser) | 1(1) | 0 | Ala:3/4^#^; CADD: 12.4(35.2); Var: VUS/B |
| *NEXN* c.53T>C | p.(Val18Ala) | 1(1) | 6; 0.016 (Other) | Ala:1/4; CADD: 23.7(35.2); Var: LB |
| ***TNNC1* c.139A>G** | **p.(Met47Val)** | **1(1)** | **0** | **Ala:2/4; CADD: 25.3(23.5); Var: VUS** |
| ***TNNC1* c.202G>A** | **p.(Gly68Ser)** | **1(1)** | **1; 00088** | **Ala:2/4; CADD: 26.8(23.5); Var: P** |
| ***TNNC1* c.304C>T** | **p.(Arg102Cys)** | **1(0)** | **3; 0.016 (East Asian)** | **Ala:3/4; CADD: 26.8(23.5); Var: VUS** |
| ***TNNT2* c.77A>G** | **p.(Asp26Gly)** | **1(1)** | **0** | **Ala:3/4; CADD: 19.7(24.3); Var: VUS** |
| ***TNNT2* c.271G>A** | **p.(Asp91Asn)** | **1(0)** | **0** | **Ala:3/4; CADD: 27.1(24.3); Var: P** |
| ***TNNT2* c.442C>T** | **p.(Arg148Trp)** | **1(1)** | **0** | **Ala:4/4; CADD: 31.0(24.3); Var: VUS** |
| ***TPM1* c.421A>G** | **p.(Met141Val)** | **1(1)** | **0** | **Ala:1/4; CADD: 21.7(21.4); Var: P** |
| ***TPM1* c.764A>T** | **p.(Asp255Val)** | **1(1)** | **0** | **Ala:2/4; CADD: 33.0(24.3); Var: VUS/LP** |
| *TTN* c.16452delA | p.Val5485* | 1(0) | 0 | Ala:n.a.; CADD: n.a; Var: LP^ |
| *TTN* c.17823delA | p.Ile5941Metfs*8 | 1(1) | 0 | Ala:n.a.; CADD: n.a; Var: LP^ |
| *TTN* c.29148delC | p.Phe9717Serfs*23 | 1(0) | 0 | Ala:n.a.; CADD: n.a; Var: P^ |
| *TTN* c.32887+1G>C |  | 1(1) | 0 | Ala:4/4^#^; CADD: 33.0(49.4); Var: P^ |
| *TTN* c.36364+2T>C |  | 1(0) | 0 | Ala:3/4^#^; CADD: 30.0(49.4); Var: P^ |
| *DSP* c.5324G>T | p.(Arg1775Ile) | 1(1) | 18; 0.055 (Other) | Ala:3/4; CADD: 25.2(27.8); Var: VUS |
| *ACTC1* c.967G>T | p.(Ala323Ser) | 2(2) | 0 | Ala:4/4; CADD: 28.5(30.0); Var: VUS/LP |
| *CSRP3* c.22G>A | p.(Ala8Thr) | 1(1) | 4; 0. 016 (Other) | Ala:1/4; CADD: 21.2(29.3); Var: VUS |
| *CSRP3* c.98C>T | p.(Thr33Met) | 1(0) | 3; 0.0026 | Ala:0/4; CADD: 22.9(29.3); Var: VUS |
| *CSRP3* c.208G>T | p.(Gly70Trp) | 1(1) | 9; 0. 022 (South Asian | Ala:3/4; CADD: 27.2(29.3); Var: B |
| *CSRP3* c.202C>G | p.(Pro86Ala) | 1(1) | 0 | Ala:4/4; CADD: 23.9(29.3); Var: VUS/LP |
| *CSRP3* c.365G>A | p.(Arg122Gln) | 1(1) | 4; 0.014 (Other) | Ala:3/4; CADD: 32.0(29.3); Var: VUS |
| *CSRP3* c.379G>A | p.(Val127Ile) | 2(1) | 9; 0.018 (African/African American) | Ala:2/4; CADD: 27.2(29.3); Var: B |
| *CSRP3 c.491_497delGGGAACT* | p.Gly164Valfs*42 | 1(0) | 0 | Ala:n.a.; CADD: n.a; Var: P^ |
| *CSRP3 c.282-5_285del* |  | 2(1) | 0 | Ala:4/4^#^; CADD: n.a.; Var: LP^ |
| *GLA* c.353G>A | p.(Arg118His) | 1(1) | 3; 0.015 (African/African American) | Ala:0/4; CADD: 13.3(23.2); Var: VUS/LP |
| *GLA* c.717A>G | p.(Ile239Met) | 1(1) | 0 | Ala:3/4; CADD: 19.1(23.2); Var: LP |
| *MYBPC3* c.175A>G | p.(Thr59Ala) | 1(1) | 0 | Ala:1/4; CADD: 0.1(24.9); Var: VUS/LP |
| *MYBPC3* c.844C>T | p.(Arg282Trp) | 1(0) | 5; 0.014 (Finnish) | Ala:2/4; CADD: 23.9(24.9); Var: VUS/LP |
| *MYBPC3* c.1021G>A | p.(Gly341Ser) | 1(1) | 14; 0.017 (Latino/Admixed American) | Ala:4/4; CADD: 28.2(24.9); Var: VUS/LP |
| *MYBPC3* c.1037G>A | p.(Arg346His) | 1(0) | 0 | Ala:0/4; CADD: 24.9(24.9); Var: VUS/LP |
| *MYBPC3* c.1372C>T | p.(Arg458Cys) | 1(1) | 10; 0.021 (African/African American) | Ala:3/4; CADD: 22.6(24.9); Var: VUS |
| *MYBPC3* c.2441_2443del | p.(Lys814del) | 1(0) | 17; 0.016 (Finnish) | Ala:n.a.; CADD: n.a; Var: VUS/LP |
| *MYBPC3* c.2815C>T | p.(Arg939Trp) | 1(0) | 9; 0.0083 (African/African American) | Ala:3/4; CADD: 26.9(24.9); Var: VUS |
| *MYBPC3* c.2828G>A | p.(Arg943Gln) | 1(0) | 4; 0.0065 (African/African American) | Ala:3/4; CADD: 31.0(24.9); Var: VUS/LP |
| *MYBPC3* c.2927C>G | p.(Pro976Arg) | 1(0) | 0 | Ala:4/4; CADD: 27.4(24.9); Var: VUS/LP |
| *MYBPC3* c.3143G>A | p.(Arg1048His) | 1(0) | 6; 0.033 (Other) | Ala:1/4; CADD: 27.0(24.9); Var: VUS |
| *MYBPC3* c.3323A>C | p.(Lys1108Thr) | 1(1) | 16; 0.014 (Other) | Ala:3/4; CADD: 26.3(24.9); Var: VUS |
| *MYBPC3* c.3358C>T | p.(Arg1120Cys) | 1(1) | 7; 0.010 (Other) | Ala:4/4; CADD: 28.9(24.9); Var: VUS |
| *MYBPC3* c.3416T>C | p.(Val1139Ala) | 1(1) | 0 | Ala:4/4; CADD: 27.3(24.9); Var: VUS/LP |
| *MYBPC3* c.3613C>T | p.(Arg1205Trp) | 1(1) | 1; 0.00088 | Ala:4/4; CADD: 31.0(24.9); Var: VUS/LP |
| ***MYH7* c.553A>T** | **p.(Thr185Ser)** | **1(1)** | **0** | **Ala:2/4; CADD: 22.2(22.7) Var: LP** |
| ***MYH7* c.1686C>A** | **p.(Asn562Lys)** | **1(1)** | **0** | **Ala:3/4; CADD: 22.8(22.7) Var: VUS/LP** |
| ***MYH7* c.1727A>G** | **p.(His576Arg)** | **1(1)** | **5; 0.0040 (African/African American)** | **Ala:3/4; CADD: 23.7(22.7) Var: P** |
| ***MYH7* c.2562G>T** | **p.(Glu854Asp)** | **1(0)** | **0** | **Ala:2/4; CADD: 22.7(22.7) Var: VUS/LP** |
| ***MYH7* c.2744T>C** | **p.(Leu915Pro)** | **1(1)** | **0** | **Ala:4/4; CADD: 28.2(22.7) Var: LP** |
| *MYH7* c.2890G>C* | p.(Val964Leu) | 4(2) | 124; 0.084 | Ala:4/4; CADD: 26.9(22.7) Var: VUS/LP |
| *MYH7* c.3134G>A* | p.(Arg1045His) | 1(1) | 10; 0.021 (East Asian) | Ala:3/4; CADD: 31.0(22.7) Var: P |
| ***MYH7* c.3169G>A** | **p.(Gly1057Ser)** | **1(1)** | **2; 0.0017** | **Ala:3/4; CADD: 27.8(22.7) Var: LP** |
| ***MYH7* c.3730A>C** | **p.(Asn1244His)** | **1(1)** | **2; 0.0017** | **Ala:3/4; CADD: 26.1(22.7) Var: VUS** |
| *MYH7* c.4817G>A* | p.(Arg1606His) | 1(1) | 11; 0.0069 | Ala:3/4; CADD: 28.1(22.7) Var: VUS |
| ***MYH7* c.5135G>A** | **p.(Arg1712Gln)** | **6(6)** | **6; 0.0046** | **Ala:3/4; CADD: 32.0(22.7) Var: P** |
| ***MYH7* c.5291T>G** | **p.(Met1764Arg)** | **1(1)** | **0** | **Ala:3/4; CADD: 25.1(22.7) Var: VUS/LP** |
| ***MYH7* c.5317C>A** | **p.(Gln1773Lys)** | **1(1)** | **0** | **Ala:3/4; CADD: 27.9(22.7) Var: VUS/LP** |
| ***MYH7* c.5762G>A** | **p.(Arg1921Gln)** | **1(1)** | **4; 0.0032 (South Asian)** | **Ala:2/4; CADD: 31.0(22.7) Var: VUS/LP** |
| ***MYL3* c.452C>T** | **p.(Ala151Val)** | **1(1)** | **0** | **Ala:4/4; CADD: 25.9(15.0) Var: P** |
| *MYL3* c.530A>G* | p.(Glu177Gly) | 1(1) | 16; 0.041 (Other) | Ala:1/4; CADD: 31.0(15.0) Var: LP |
| *PRKAG2* c.253C>T | p.(Pro85Ser) | 1(0) | 2; 0.0018 | Ala:0/4; CADD: 19.1(24.1) Var: VUS |
| *PRKAG2* c.455G>A | p.(Arg152His) | 2(1) | 13; 0.013 (Other) | Ala:1/4; CADD: 29.7(24.1) Var: B |
| *TNNC1* c.210C>T | p.(Gly70Gly) | 2(1) | 32; 0.023 | Ala:4/4^#^; CADD: 13.7(23.5); Var: LB |
| *TNNC1* c.248G>A | p.(Arg83Gln) | 1(1) | 2; 0.0099 (Ashkenazi Jewish) | Ala:3/4; CADD: 27.7(24.3) Var: VUS/LP |
| ***TNNT2* c.517G>C** | **p.(Glu173Gln)** | **1(1)** | **0** | **Ala:3/4; CADD: 27.0(24.3); Var: VUS/LP** |
| ***TNNT2* c.865A>G** | **p.(Lys289Glu)** | **1(1)** | **0** | **Ala:2/4; CADD: 25.8(24.3); Var: VUS** |
| ***TPM1* c.89C>T** | **p.(Thr30Ile)** | **1(1)** | **0** | **Ala:1/4; CADD: 12.9(24.3); Var: VUS/LP** |
| DCM |  |  |  |  |
| ACM |  |  |  |  |
| HCM |  |  |  |  |

The number of cases with a specific variant identified is indicated, and, between brackets, the number of cases in which prioritisation/reclassification would potentially lead to a change in diagnostic outcome (*i.e.* in some cases respective patient is already carrier of another (L)P). Indicated in bold are variants that have been reclassified to LP. *Variants were prioritised but not reclassified to LP at this stage as they were identified too often in the general population and did not apply to rule PM2. gnomAD: AC (allele count); AF (allele frequency in %) = highest frequency of variant in NFE gnomAD (when highest frequency was found in other cohort this is specifically indicated); Path Pred = predictions of pathogenicity via (1) the Alamut software; indicated as Ala:../..: number of prediction programs predicting pathogenicity (Align AGVD: ≥Class C45, SIFT: Deleterious, Mutation taster: Deleterious and PolyPhen (HumVar): possibly or probably pathogenic) or splice site recognition^#^ (SpliceSiteFinder like, MaxEntScan, NNSPLICE, GeneSplicer)/total of prediction programs offered by Alamut; (2) CADD (<https://cadd.gs.washington.edu/snv>): CADD score prediction variant (Path CADD score for gene), and (3) Varsome prediction (<https://varsome.com>) (including pathogenic computational verdict based on 12 pathogenic predictions from BayesDel_addAF, DANN, DEOGEN2, FATHMM-MKL, LIST-S2, M-CAP, MutationTaster, SIFT, EIGEN, MVP, MutationAssessor and PrimateAI); ^note that Varsome automatically uses the ACMG rule PVS1 for variants resulting in a premature stop codon and thus leading to classification (L)P, however not for every gene (region) such association with disease is established; n.a.: not applicable.

**Supplemental Table 2b. VUSs in genes/gene regions with EF≥0.9 and reclassified to LP within specific CM subtypes in our patient cohort**

| Variant | Protein change | Cases/VUS | Additional information pathogenicity* |
| --- | --- | --- | --- |
| *TNNC1* c.139A>G | p.(Met47Val) | 1(1) | Unique in our cohort of patients |
| *TNNC1* c.202G>A | p.(Gly68Ser) | 1(1) | Unique un our cohort of patients; PS4_Supporting^#^: our patient and PMID: 33179204 |
| *TNNC1* c.304C>T | p.(Arg102Cys) | 1(0) | PS4_Moderate^#^: currently in 2 patients in our cohort, PMID 33179204, 30188508, 26341255, 31983221, 33658040 and 2 entries ClinVar~. |
| *TNNT2* c.77A>G | p.(Asp26Gly) | 1(1) | Unique in our cohort of patients |
| *TNNT2* c.271G>A | p.(Asp91Asn) | 1(0) | Unique in our cohort of patients; PS4_Supporting^#^: our patient and 3 entries ClinVar. |
| *TNNT2* c.442C>T | p.(Arg148Trp) | 1(1) | Unique in our cohort; PS4_Moderate^#^: our patient, PMID 29540472, 30847666, 32880476, 32041989, and 3 entries ClinVar. |
| *TPM1* c.421A>G | p.(Met141Val) | 1(1) | Unique in our cohort; PS4_Supporting^#^: our patient and 1 entry ClinVar; PM5^#^^: PMID 24503780, 27532257, 2458740, 33019804, 31308319 |
| *TPM1* c.764A>T | p.(Asp255Val) | 1(1) | Unique in our cohort of patients |
| *MYH7* c.553A>T | p.(Thr185Ser) | 1(1) | Unique in our cohort of patients |
| *MYH7* c.1686C>A | p.(Asn562Lys) | 1(1) | PS4_Supporting^#^: currently in 4 patients (2 affected sibs) in our cohort and 1 entry ClinVar. |
| *MYH7* c.1727A>G | p.(His576Arg) | 1(1) | Unique in our cohort, identified in 2 affected sibs; PS4_Moderate^#^: our family, PMID 15856146, 19666645, 24111713, 20646679, 27247418, 27532257, 30868567, 30847666 31447099 32894683, 33673806 and 9 entries ClinVar |
| *MYH7* c.2562G>T | p.(Glu854Asp) | 1(0) | Unique in our cohort, identified in 2 affected sibs; PS4_Supporting^#^: our family and 2 entries ClinVar |
| *MYH7* c.2744T>C | p.(Leu915Pro) | 1(1) | Unique in our cohort; PS4_Supporting^#^: our patient and 2 entries ClinVar; |
| *MYH7* c.3169G>A | p.(Gly1057Ser) | 1(1) | PS4^#^: currently in 2 patients in our cohort, PMID 15358028, 24510615, 27532257, 24510615, 30297972, 30847666, 31513939, 328804762, 29540472 29300372, 32894683, and 6 entries ClinVar; PP5 (ClinGen Cardiomyopathy Variant Curation Expert Panel): classified as LP |
| *MYH7* c.3730A>C | p.(Asn1244His) | 1(1) | Unique in our cohort; PS4_Supporting^#^: our patient and 3 entries ClinVar; |
| *MYH7* c.5135G>A | p.(Arg1712Gln) | 6(6) | PS4^#^: currently in 8 patients in our cohort, PMID 21511876, 23054336, 23785128, 25892673, 25351510, 27247418, 27532257, 27688314, 28771489, 28193612, 2879411, 29300372, 29661763, 30022097, 30847666, 31308319, 32894683, 33673806 and 21 entries ClinVar; PP5 (ClinGen Cardiomyopathy Variant Curation Expert Panel) and classified as P; PM5^#^^: PMID 15483641, 21511876, 23197161, 24498601, 31130376, 32894683 |
| *MYH7* c.5291T>G | p.(Met1764Arg) | 1(1) | Unique in our cohort of patients |
| *MYH7* c.5317C>A | p.(Gln1773Lys) | 1(1) | Unique in our cohort of patients; PS4_Supporting^#^: our patient, PMID: 29121657 and 1 entry ClinVar |
| *MYH7* c.5762G>A | p.(Arg1921Gln) | 1(1) | Unique in our cohort of patients; PS4_Supporting^#^: our patient, PMID: 32746448 and 4 entries ClinVar; PM5^#^^: PMID 31983221 |
| *MYL3* c.452C>T | p.(Ala151Val) | 1(1) | Unique in our cohort, identified in 2 affected sibs + affected uncle obligate carrier; PS4_Moderate^#^: our patient, PMID 29661763, 31513939 and 2 entries ClinVar; PP1 (2 affected sibs + affected uncle) |
| *TNNT2* c.517G>C | p.(Glu173Gln) | 1(1) | Unique in our cohort, identified in 2 affected sibs PS4_Supporting^#^: our family and 1 entry ClinVar; PM5^#^^: PMID 7898523, 27532257, 22112859, 22144547, including functional proof: 10617660, 24480310, 22579624, 14722098, 24367593 |
| *TNNT2* c.865A>G | p.(Lys289Glu) | 1(1) | Unique in our cohort of patients; PS4_Supporting^#^: our patient and 1 entry ClinVar |
| *TPM1* c.89C>T | p.(Thr30Ile) | 1(1) | PS4_Supporting^#^: currently in 2 patients in our cohort |
| DCM |  |  |  |
| HCM |  |  |  |

The number of cases with a specific variant identified is indicated, and, between brackets, the number of cases in which reclassification would potentially lead to a change in diagnostic outcome (*i.e.,* in some cases respective patient is already carrier of another (L)P). Indicated in red are variants in genes/gene regions with EF≥0.95 and in orange variants in genes/gene regions with 0.9≤EF<0.95). *In addition to PM2, PM1_strong or PM1_moderate, PP3 and PP4; ^#^(adapted) ACMG criteria (Kelly et al., 2018; Richards et al., 2015): PS4_Moderate: Variant identified in ≥6 probands with consistent phenotypes; PS4_Supporting: Variant identified in ≥2 probands with consistent phenotypes; PM5: Missense change at an amino acid residue where a different missense change previously established as pathogenic; PS4: Variant identified in ≥15 probands with consistent phenotypes; PP5: Reputable source recently reports variant as pathogenic; PP1: Variant segregates in ≥3 meioses; ~Note that (1) overlap may exist between publications and ClinVar entries and (2) our own submission (when appropriate) to ClinVar not included; ^Note that the criteria referenced refers to variants established as pathogenic: in this case pathogenicity of these are not necessarily yet established. PMID = PubMed ID.

**Supplemental Table 3. VUSs detected in genes with mis_z>3 and/or pLI>0.9 according to gnomAD**

| **Variant** | **Protein change** | **Cases** |
| --- | --- | --- |
| *ABCC9* c.2023A>G | p.(Thr675Ala) | 1 |
| *ABCC9* c.2324C>A | p.(Pro775His) | 1 |
| *ABCC9* c.2674A>G | p.(Arg892Gly) | 1 |
| *ABCC9* c.2784C>G | p.(Arg928Glu) | 1 |
| *ABCC9* c.3221A>G | p.(Asn1074Ser) | 1 |
| *RYR2* c.892C>T | p.(Arg298Cys) | 1 |
| *RYR2* c.1574C>G | p.(Ser525Cys) | 1 |
| *RYR2* c.1939C>T | p.(Arg647Cys) | 3 |
| *RYR2* c.2207G>T | p.(Cys736Phe) | 1 |
| *RYR2* c.2617G>A | p.(Val873Met) | 1 |
| *RYR2* c.2717C>T | p.(Pro906Leu) | 1 |
| *RYR2* c.3152G>A | p.(Arg1051His) | 1 |
| *RYR2* c.5021A>G | p.(His1674Arg) | 1 |
| *RYR2* c.6079G>A | p.(Gly2027Arg) | 1 |
| *RYR2* c.6593G>A | p.(Arg2198His) | 1 |
| *RYR2* c.6952A>G | p.(Asn2318Asp) | 1 |
| *RYR2* c.8147A>T | p.(Lys2716Ile) | 3 |
| *RYR2* c.8968C>T | p.(Leu2990Phe) | 1 |
| *RYR2* c.9454C>T | p.(Arg3152Cys) | 1 |
| *RYR2* c.10031C>T | p.(Ala3344Val) | 1 |
| *RYR2* c.10100A>G | p.(Tyr3367Cys) | 1 |
| *RYR2* c.10738C>T | p.(Gln3580*) | 1 |
| *RYR2* c.10790G>T | p.(Arg3597Met) | 1 |
| *RYR2* c.10933G>A | p.(Ala3645Thr) | 1 |
| *RYR2* c.12341G>A | p.(Arg4114Gln) | 2 |
| *RYR2* c.12488C>A | p.(Pro4163His) | 1 |
| *RYR2* c.12633G>C | p.(Leu4211Phe) | 1 |
| *RYR2* c.12943G>A | p.(Gly4315Arg) | 1 |
| *RYR2* c.14387G>A | p.(Ser4796Asn) | 1 |
| *ACTC1* c.632T>G | p.(Val211Gly) | 1 |
| *MYH7* c.266A>G | p.(Asp89Gly) | 1 |
| *MYH7* c.2945T>C | p.(Met982Thr) | 1 |
| *MYH7* c.4076G>A | p.(Arg1359His) | 1 |
| *MYH7* c.4187G>A | p.(Arg1396Gln) | 1 |
| *MYH7* c.5779A>T | p.(Ile1927Phe) | 1 |
| *TBX20* c.1272_1273delinsA | p.(Tyr424*) | 1 |
| *TBX20* c.146dupC | p.(Ser50Valfs*8) | 1 |
| TOTAL |  | 42 |

List of VUSs identified with mis_z>3 or pLI>0.9 in our cohort after filtering on variants in genes not analyzed for CE, but within DCM, HCM and/or ARVC cardiomyopathy subtypes. Number of cases carrying a particular variant is indicated.

**Supplemental Figure 1: VUS classification/prioritisation flow chart**


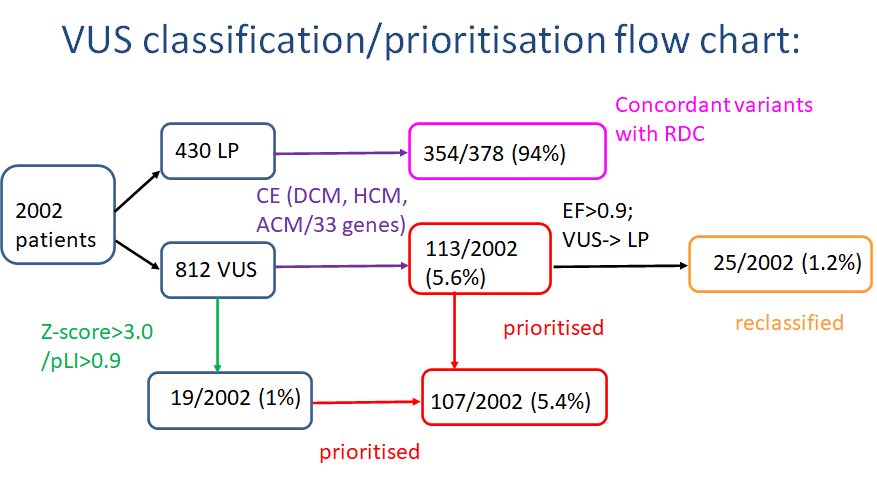


Using routine diagnostic criteria, variants detected from our cohort of 2002 patients were classified. Applying CE and EFs scores (i.e., constraint metrics) based evaluations confirmed 94% of classified (L)P variants compared to RDC. CE from 33 genes in DCM, HCM and ACM was applied to select for VUSs [N=812] in our cohort for potential prioritisation leading to such VUSs [N=113] identified in 5.6% (113) of our patients. Of these, VUSs who had an EF>0.9 were reclassified to LP making up 1.2% of the cohort (25 patients). Using z-score>3 and pLI>0.9 criteria on VUSs in genes not included in the CE/EF selection, 19 patients were identified with additional VUSs for prioritisation. Total patients with VUSs prioritised lead to 5.4% of the cohort (107 patients).
